# Identification of miRNAs and their target genes in developing soybean seeds by deep sequencing

**DOI:** 10.1186/1471-2229-11-5

**Published:** 2011-01-10

**Authors:** Qing-Xin Song, Yun-Feng Liu, Xing-Yu Hu, Wan-Ke Zhang, Biao Ma, Shou-Yi Chen, Jin-Song Zhang

**Affiliations:** 1 State Key Laboratory of Plant Genomics, Genome Biology Center, Institute of Genetics and Developmental Biology, Chinese Academy of Sciences, Beijing 100101, PR China

## Abstract

**Background:**

MicroRNAs (miRNAs) regulate gene expression by mediating gene silencing at transcriptional and post-transcriptional levels in higher plants. miRNAs and related target genes have been widely studied in model plants such as *Arabidopsis *and rice; however, the number of identified miRNAs in soybean (*Glycine max*) is limited, and global identification of the related miRNA targets has not been reported in previous research.

**Results:**

In our study, a small RNA library and a degradome library were constructed from developing soybean seeds for deep sequencing. We identified 26 new miRNAs in soybean by bioinformatic analysis and further confirmed their expression by stem-loop RT-PCR. The miRNA star sequences of 38 known miRNAs and 8 new miRNAs were also discovered, providing additional evidence for the existence of miRNAs. Through degradome sequencing, 145 and 25 genes were identified as targets of annotated miRNAs and new miRNAs, respectively. GO analysis indicated that many of the identified miRNA targets may function in soybean seed development. Additionally, a soybean homolog of Arabidopsis SUPPRESSOR OF GENE SLIENCING 3 (*AtSGS3*) was detected as a target of the newly identified miRNA Soy_25, suggesting the presence of feedback control of miRNA biogenesis.

**Conclusions:**

We have identified large numbers of miRNAs and their related target genes through deep sequencing of a small RNA library and a degradome library. Our study provides more information about the regulatory network of miRNAs in soybean and advances our understanding of miRNA functions during seed development.

## Background

MicroRNAs (miRNAs) are endogenous ~21-nt noncoding RNAs derived from single-stranded RNA precursors that can form stem-loop structures [1,2]. MiRNA was first identified in *Caenorhabditis elegans *and subsequently found in almost all eukaryotes [3]. In higher plants, miRNAs play important roles in different developmental stages by mediating gene silencing at transcriptional and post-transcriptional levels [4-6]. Soybean is the most widely planted oil crop in the world; however, the regulation of its seed development is not well studied. The roles of miRNAs in soybean seed development remain largely unknown. Therefore, identification of new miRNAs and elucidation of their functions in seed development will help us understand the regulation of soybean lipid synthesis. Recently, the soybean genome sequence has been finished [7], which will greatly advance biological research on soybeans.

Although many soybean miRNAs were identified in previous research [8-10], the number of miRNAs known in soybean is still very small and considerably lower than that in Arabidopsis or rice. Most identified soybean miRNAs are of high abundance and conserved in many species; however, low-abundance and species-specific miRNAs may play important roles in soybean-specific processes. Generally, it is not easy to get information on these miRNAs by conventional methods. Recently, next-generation sequencing technology has been developed and widely applied to genomic studies such as gene expression pattern analysis, genome sequencing and small RNA sequencing. Because of its ultra high-throughput, many new miRNAs with low abundance could be identified using this technology.

To date, the majority of miRNA targets in soybean were predicted by bioinformatics approaches, and only a small portion were experimentally validated. A high-throughput degradome library sequencing technology has been developed for global identification of targets of miRNAs in Arabidopsis, rice and grapevine [11-18]. To detect new miRNAs participating in soybean seed development and to identify targets of soybean miRNAs globally, a small RNA library and a degradome library using RNAs from developing soybean seeds were constructed and sequenced by a Solexa analyzer. Each library generated more than 6 million short reads, and 26 new miRNAs were identified, of which 17 miRNAs belong to new families and 9 miRNAs belong to conserved families. A total of 170 genes sliced by small RNAs were detected via degradome library sequencing. Among these, 64 genes were reproduction-related genes, and the corresponding miRNAs may have a function in soybean seed development.

## Results

### Overview of small RNA library sequencing

**Figure 1:**
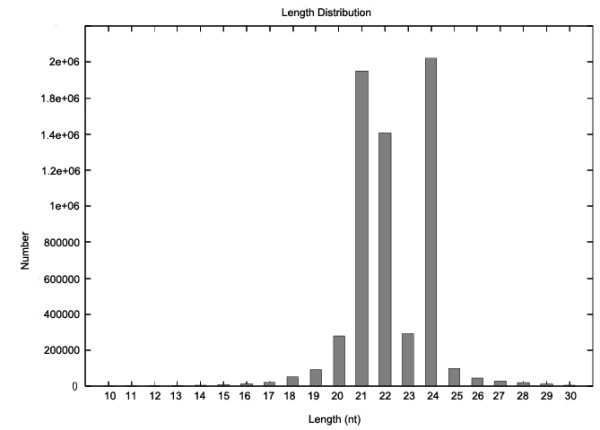
**Distribution of Solexa reads in the soybean small RNA library**. Solexa reads with 21, 22, or 24 nucleotides were the most enriched in total small RNA sequences.

The soybean small RNA library was constructed using RNAs obtained from seeds of 15-day-old after flowering and sequenced by Solexa SBS technology. We obtained more than 6 million raw reads, ranging from 18 to 30 nucleotides in length. As seen in Figure 1, the highest abundance was found for sequences with 21, 22 and 24 nucleotides (nt). After removal of low quality reads and adapter contaminants, 2,145,586 unique reads were collected and 1,495,099 (69.8%) sequences were perfectly mapped to the soybean genome using SOAP2 software (Table 1) [19]. Small RNAs were analyzed by BLAST against the known noncoding RNAs (rRNA, tRNA, snRNA, snoRNA) deposited in the Rfam and NCBI Genbank databases [20]. 25,944 distinct small RNAs belonging to these categories were removed to avoid degradation contamination. The remaining reads were used to identify the conserved and new miRNAs.

**Table 1:** Different categories of small RNAs by deep sequencing

Category	Unique reads	Total reads
All reads	2,145,586	5,908,211
Match genome^a^	1,495,099	4,790,766
Known miRNAs^b^	1,695	677,062
Rfam^c^	25,944	450,869
Unannotated	1,467,460	3,662,835

^a^Genome sequences downloading from Glyma1 assembly

^b^Known miRNAs deposited at miRBase database

^c^Rfam including rRNA, tRNA, snRNA and snoRNA

### Prediction and validation of new miRNAs

**Figure 2:**
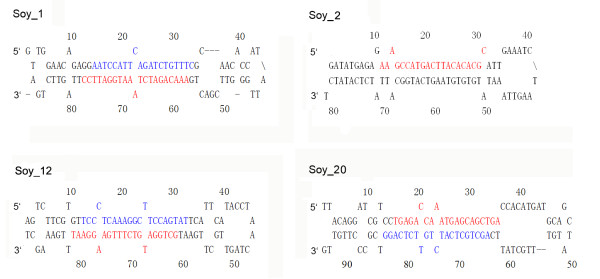
**Predicted RNA hairpin structures of new miRNA precursors**. Precursor structures of 4 newly identified soybean miRNAs (Soy_1, Soy_2, Soy_12, and Soy_20) were predicted by MFOLD pipeline. Mature miRNA and miRNA star sequences are highlighted in red and blue, respectively. The numbers along the structure are nucleotide sites from the 5' end of the pre-miRNA sequence.

In total, 207 soybean miRNAs were annotated in the latest miRBase database [21,22], and most of these were identified by small RNA library sequencing. In this study, 55 annotated miRNAs were detected in a seed small RNA library. The remaining 152 miRNAs, mostly soybean specific, were not detected, possibly because of low expression levels or spatial expression pattern. Twenty-six new soybean miRNAs not previously reported were identified by bioinformatic analysis. These new miRNAs were named temporarily in the form of Soy_number, e.g., Soy_1 (Table S1 in Additional File 1). Among the 26 new miRNAs, 17 miRNAs belonged to new families that had never been found in eukaryotes (Table S1 in Additional File 1). All precursors of new miRNAs had regular stem-loop structures, and four of these, Soy_1, Soy_2, Soy_12 and Soy_20, were presented in Figure 2. These RNA structures were predicted by MFOLD software and checked manually [23].

**Figure 3:**
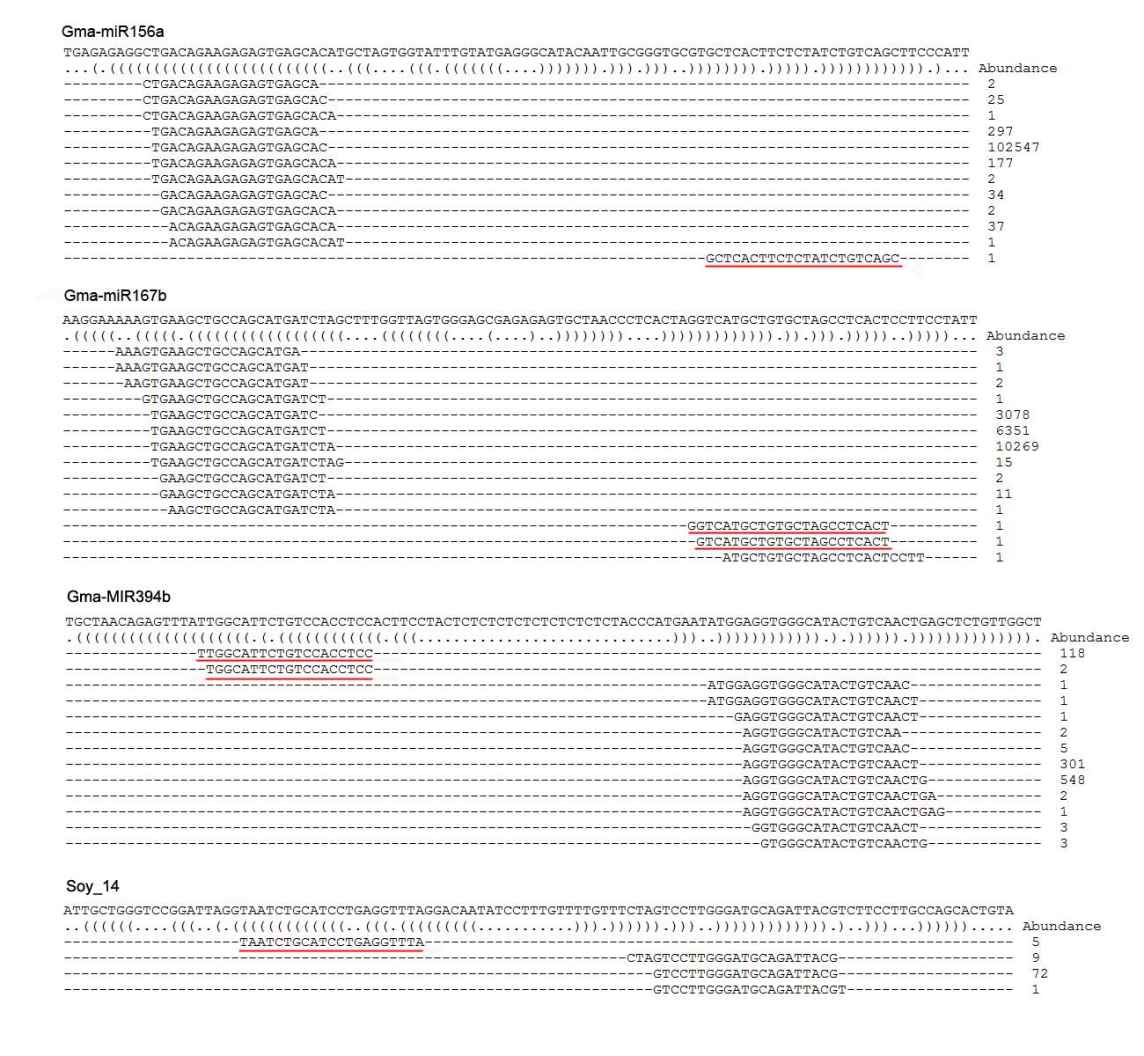
**Diversification of mature miRNA production from miRNA precursors**. Detected diverse isoforms of three conserved and one new mature miRNAs from soybean are shown. MiRNA star sequences are underlined in red. "Abundance" is the detected number of reads in small RNA library sequencing.

Forty-six miRNA-star sequences (miRNA*), the complementary strands of functional mature miRNA, were also detected in this study (Table S1 in Additional File 1). These sequences are rarely found via conventional sequencing because of their quick degradation in cells. The detection of miRNA* represented further evidence for the existence of mature miRNAs. The miRNA* sequences for 38 known miRNAs and 8 new miRNAs were discovered (Figure 2, 3; Table S1 in Additional File 1). Soy_13 is the star strand of Soy_25, which belongs to the family of miR2118 [24]. Gso-miR2118 has been validated in wild soybean by northern blot in previous research [24]. In our study, Soy_13 was detected 3 times more than Soy_25 by Solexa sequencing (Table S1 in Additional File 1). Therefore, Soy_13 may be also a functional miRNA in soybean, not a miRNA* of Soy_25. In Figure 2, miRNA mature sequences and miRNA* sequences in miRNA precursors are highlighted using different colors. Their locations relative to RNA loops in precursors were not invariable. Large-scale sequencing allowed us to identify many mature miRNA variants, which represent some differences in the 5' and/or 3' ends of mature miRNA sequences (Figure 3).

**Figure 4:**
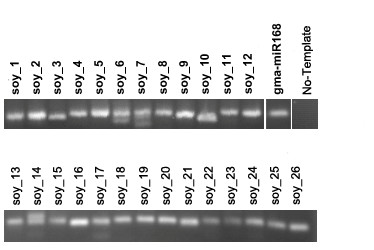
**Stem-loop RT-PCR for identified new miRNAs**. In total, 26 new miRNAs were confirmed by stem-loop RT-PCR with 40-cycle-amplification. The sizes of PCR products were around ~60 bp. Gma-miR168: the positive control; No Template: no RNA was added as a template in the RT reaction.

To validate the predicted new miRNAs, stem-loop RT-PCR was performed to examine their expression in soybean seeds [25]. Primers used in stem-loop RT-PCR are listed in Table S2 in Additional File 2. All of the 26 predicted miRNAs were found to be expressed in soybean seeds (Figure 4). The gma-miR168 was amplified as a positive control (Figure 4).

### Soybean seed degradome library construction and sequencing

To identify the target genes of miRNAs in the soybean transcriptome, the widely adopted technology of degradome library sequencing was applied in this study [11-16]. MiRNAs mediate gene silencing by two mechanisms: mRNA cleavage and translation repression. In higher plants, miRNAs slice mRNAs to regulate gene expression in most cases [1,2,11]. MiRNA-directed cleavage leaves a free 5' phosphate at the 3' fraction of the sliced genes. Through poly(A) RNA purification, we constructed a 5' uncapped mRNA library. The transcriptome-wide degradome information can be collected through high-throughput sequencing. We constructed a soybean seed degradome library and obtained more than 15 million raw reads with 99% of sequences having 20 or 21 nt by Solexa sequencing. After quality filtration and adapter removal, we obtained 1,662,975 unique reads, of which 1,062,557 (64%) were perfectly matched to the soybean genome (Table 2). However, only 663,641 (40%) reads could be mapped to a single position in the soybean genome. Interestingly, 308,578 (18%) reads had two hits in the genome. We further used the published Williams 82 cDNA database as the template to map clean reads. In total, 1,044,162 unique reads were mapped to the soybean cDNAs, indicating the high quality of the present degradome library (Table 2). The reads that mapped to soybean cDNAs were subjected to further analysis.

**Table 2:** Summary of degradome reads mapping statistics

Raw reads	Unique reads	Genome mapped reads	Reads with single hit to genome	**cDNA mapped reads**^**a**^
15168792	1662975	1062557	663,641	1044162

^a^cDNA sequences downloaded from Glyma1 assembly

### Identification and classification of targets for annotated miRNAs

Compared to other mRNA degradation mechanisms, miRNA mediated mRNA cleavage possesses special features. The sliced region of the mRNA should be complementary to the miRNA sequence, and the cleavage site is usually between the 10^th ^and 11^th ^nucleotides from the 5' end of the miRNA. These features were used to identify targets of miRNAs. We first extracted 15 nt upstream and downstream of 5' soybean cDNAs sequences mapped by degradome reads to generate 30 nt target signatures as "t-signature" [12]. These signatures were collected to find miRNA targets using CleaveLand pipeline [18]. According to the abundance of miRNA-complemented signatures relative to other signatures mapped to mRNAs, the identified targets could be sorted into 4 classes. The targets with only miRNA-directed cleavages were classified as Class I. In Class II, the cleavage signature abundance was mostly enriched among all signatures. The abundance of cleavage signatures was higher than the median in Class III targets. The rest, with a low abundance of cleavage signatures, were grouped as Class IV. Because the abundance of miRNA-directed cleavage targets in Class I and Class II was much higher than other signatures, the targets in these two classes could have low false discovery rates and be more accurate. All identified miRNA targets were classified according to these criteria.

To date, 207 soybean annotated miRNAs have been deposited in the miRBase database. Few miRNA targets have been validated by experimental methods [8-10]. In our study, 126 targets of 19 evolutionarily conserved miRNA families were identified (Table 3). Only 9 soybean-specific miRNA families were found to silence 19 genes (Table 3; the miRNAs designated by ^a^). It should be noted that many targets of a single conserved miRNA are in pairs with very similar sequences, and the gma-miR156, gma-miR160, gma-miR164, gma-miR166, gma-miR172 and gma-miR396 had at least 10 targets, with the gma-miR396 having more than 20 targets (Table 3). On the other hand, the soybean-specific miRNAs appear to have only a limited number of targets.

**Table 3:** Identified targets of known miRNAs in soybean

miRNA	Target gene	Target annotation	Class	Abundance(TP10M)	cleavage site(nt)	target site location
gma-miR156	Glyma04g37390#	SBP domain protein	I	39	938	5'UTR
	Glyma06g17700#	SBP domain protein	I	39	1185	CDS
	Glyma05g00200*	SBP domain protein	I	42	1202	CDS
	Glyma04g32070*	SBP domain protein	II	42	130	3'UTR
	Glyma17g08840*	SBP domain protein	I	42	1011	CDS
	Glyma05g38180#	SBP domain protein	I	33	1356	CDS
	Glyma08g01450#	SBP domain protein	I	33	1154	CDS
	Glyma18g00890	SBP domain protein	II	75	249	CDS
	Glyma12g27330	SBP domain protein	II	17	621	5'UTR
	Glyma11g36980	SBP domain protein	II	47	1223	CDS
	Glyma02g30670	SBP domain protein	I	109	688/689	CDS
	Glyma18g36960	SBP domain protein	I	14	723	CDS
	Glyma02g13370	SBP domain protein	I	173	1219/1220	CDS
gma-miR159	Glyma15g35860#	MYB family transcription factor	II	27	937	CDS
	Glyma13g25720#	MYB family transcription factor	II	27	838	CDS
	Glyma20g11040	MYB family transcription factor	I	17	918	CDS
gma-miR160	Glyma11g20490#	Auxin response factor	II	134	1510	CDS
	Glyma10g35480#	Auxin response factor	I	134	740	CDS
	Glyma12g08110#	Auxin response factor	II	134	1501	CDS
	Glyma13g20370*	Auxin response factor	I	177	1670	CDS
	Glyma10g06080*	Auxin response factor	I	177	1355	CDS
	Glyma13g02410	Auxin response factor	I	74	1280	CDS
	Glyma14g33730	Auxin response factor	I	29	1184	CDS
	Glyma19g36570	Auxin response factor	II	807	652	CDS
	Glyma04g43350	Auxin response factor	II	43	1337	5'UTR
	Glyma13g40030	Auxin response factor	II	67	1277	CDS
	Glyma20g32040	Auxin response factor	I	19	1313	CDS
	Glyma12g29720	Auxin response factor	I	25	1626	CDS
gma-miR162	Glyma12g35400*	embryo-related protein	IV	13	995	CDS
	Glyma13g35110*	embryo-related protein	IV	13	963	CDS
gma-miR164	Glyma17g10970#	NAC family transcription factor	I	750	795	CDS
	Glyma05g00930#	NAC family transcription factor	II	750	751	CDS
	Glyma06g21020#	NAC family transcription factor	I	750	741	CDS
	Glyma04g33270#	NAC family transcription factor	I	750	634	CDS
	Glyma13g34950*	NAC family transcription factor	I	153	747	CDS
	Glyma12g35530*	NAC family transcription factor	II	153	712	CDS
	Glyma15g40510#	NAC family transcription factor	II	34	730	CDS
	Glyma08g18470#	NAC family transcription factor	II	34	731	CDS
	Glyma12g26190	NAC family transcription factor	I	87	778	CDS

miRNA	Target gene	Target annotation	Class	Abundance(TP10M)	cleavage site(nt)	target site location

gma-miR164	Glyma06g35660	NAC family transcription factor	I	24	811	CDS
gma-miR166	Glyma15g13640#^b^	HD-ZIP transcription factor	II	273	568/570	CDS
	Glyma08g21610#^b^	HD-ZIP transcription factor	II	235	898	CDS
	Glyma04g09000#	HD-ZIP transcription factor	II	273	93-95	CDS
	Glyma07g01950#	HD-ZIP transcription factor	II	273	618/620	CDS
	Glyma08g21620#	HD-ZIP transcription factor	II	273	789/791	CDS
	Glyma07g01940#	HD-ZIP transcription factor	II	273	919/921	CDS
	Glyma06g09100#^b^	HD-ZIP transcription factor	II	273	567/569	CDS
	Glyma05g30000*	HD-ZIP transcription factor	II	59	1041	CDS
	Glyma08g13110*	HD-ZIP transcription factor	II	59	571	CDS
	Glyma09g02750*	HD-ZIP transcription factor	II	59	568	CDS
	Glyma12g08080#	HD-ZIP transcription factor	II	160	1239	CDS
	Glyma11g20520#	HD-ZIP transcription factor	II	160	605/607	CDS
gma-miR167	Glyma15g09750*	Auxin response factor	II	159	2444	CDS
	Glyma13g29320*	Auxin response factor	II	159	3359	CDS
	Glyma05g27580#	Auxin response factor	II	86	2288	CDS
	Glyma08g10550#	Auxin response factor	II	86	2477	CDS
	Glyma18g05330	Auxin response factor	II	54	2880	CDS
	Glyma15g00770	zinc finger family protein	I	112	1815	5'UTR
	Glyma02g40650	Auxin response factor	II	76	2924	CDS
gma-miR168	Glyma16g34300#	AGO protein	II	74	534	CDS
	Glyma09g29720#	AGO protein	II	74	409	CDS
gma-miR169	Glyma08g45030*	NUCLEAR FACTORY	II	31	1294	5'UTR
	Glyma18g07890*	NUCLEAR FACTORY	I	31	957	CDS
	Glyma17g05920#	NUCLEAR FACTORY	I	64	1262	5'UTR
	Glyma13g16770#	NUCLEAR FACTORY	II	64	1022	CDS
	Glyma09g07960*	NUCLEAR FACTORY	II	33	931	5'UTR
	Glyma15g18970*	NUCLEAR FACTORY	II	33	981	5'UTR
	Glyma19g38800	NUCLEAR FACTORY	I	23	1385	5'UTR
gma-miR171	Glyma08g08590#	polyubiquitin protein	IV	13	195	CDS
	Glyma05g25610#	polyubiquitin protein	IV	13	187	CDS
	Glyma09g04950	TCP family transcription factor	IV	19	39	3'UTR
gma-miR172	Glyma19g35560*	heat shock cognate protein	IV	47	282	CDS
	Glyma03g32850*	heat shock cognate protein	IV	47	480	CDS
	Glyma15g04930#	AP2 transcription factor	II	425	1279	CDS
	Glyma13g40470#	AP2 transcription factor	II	348	1798	CDS
	Glyma11g15650#	AP2 transcription factor	II	425	1811	5'UTR
	Glyma12g07800#	AP2 transcription factor	II	425	1763	CDS
	Glyma01g39520*	AP2 transcription factor	II	44	1709	CDS
	Glyma11g05720*	AP2 transcription factor	II	44	1777	CDS
	Glyma19g36200#	AP2 transcription factor	II	111	1447	CDS
	Glyma03g33470#	AP2 transcription factor	II	111	1243	CDS

miRNA	Target gene	Target annotation	Class	Abundance(TP10M)	cleavage site(nt)	target site location

gma-miR172	Glyma17g18640	AP2 transcription factor	III	26	1973	CDS
	Glyma02g09600	AP2 transcription factor	II	78	1469	CDS
	Glyma05g27370#	TCP family transcription factor	II	109	922	5'UTR
gma-miR319	Glyma13g29160#	TCP family transcription factor	II	112	2078	CDS
	Glyma08g10350#	TCP family transcription factor	II	109	1172	CDS
	Glyma15g09910#	TCP family transcription factor	II	112	959	CDS
	Glyma13g34690*	TCP family transcription factor	II	195	656	CDS
	Glyma12g35720*	TCP family transcription factor	II	195	1223	CDS
	Glyma14g06680#	Plasma membrane intrinsic protein	III	49	935	CDS
	Glyma02g42220#	Plasma membrane intrinsic protein	III	49	1029	5'UTR
	Glyma13g36840*	TCP family transcription factor	II	73	1220	CDS
	Glyma12g33640*	TCP family transcription factor	II	73	740	CDS
gma-miR390	Glyma15g14670	expressed protein	IV	14	569	CDS
gma-miR393	Glyma03g36770#	Auxin signaling F-BOX protein	II	65	1750	CDS
	Glyma19g39420#	Auxin signaling F-BOX protein	II	65	1751	CDS
	Glyma16g05500*	Auxin signaling F-BOX protein	II	46	2279	CDS
	Glyma19g27280*	Auxin signaling F-BOX protein	II	46	2207	CDS
	Glyma10g02630#	Auxin signaling F-BOX protein	IV	14	2166	CDS
	Glyma02g17170#	Auxin signaling F-BOX protein	IV	14	1741	CDS
gma-miR394	Glyma01g06230*	NADP+	IV	24	42	CDS
	Glyma06g01850*	NADP+	IV	24	588	CDS
gma-miR396	Glyma03g02500#	Growth regulating factor	I	57	550	CDS
	Glyma01g34650#	Growth regulating factor	I	57	128	CDS
	Glyma09g34560*	Growth regulating factor	I	361	323	CDS
	Glyma01g35140*	Growth regulating factor	II	361	290	CDS
	Glyma07g04290#	Growth regulating factor	II	117	473	CDS
	Glyma16g00970#	Growth regulating factor	I	117	353	CDS
	Glyma13g16920*	Growth regulating factor	I	77	742	CDS
	Glyma17g05800*	Growth regulating factor	I	77	422	CDS
	Glyma09g07990*	Growth regulating factor	II	77	380	CDS
	Glyma11g11820#	Growth regulating factor	I	279	386	CDS
	Glyma11g01060#	Growth regulating factor	II	279	349	CDS
	Glyma12g01730#	Growth regulating factor	II	279	504	CDS
	Glyma01g44470#	Growth regulating factor	I	279	428	CDS
	Glyma17g35090*	Growth regulating factor	II	1007	913	CDS
	Glyma17g35100*	Growth regulating factor	II	1007	724	CDS
	Glyma14g10090*	Growth regulating factor	II	1007	704	CDS
	Glyma04g40880	Growth regulating factor	I	46	233	CDS
	Glyma06g13960	Growth regulating factor	II	46	831	CDS
	Glyma13g22840	Growth regulating factor	IV	13	282	3'UTR
	Glyma14g10100	Growth regulating factor	II	373	711	CDS
	Glyma15g19460	Growth regulating factor	II	69	347	CDS

miRNA	Target gene	Target annotation	Class	Abundance(TP10M)	cleavage site(nt)	target site location

gma-miR398	Glyma15g13870	MtN19-like protein	I	15	172	CDS
	Glyma14g39910	Serine-type endopeptidase	II	87	1370	CDS
	Glyma19g42890	Copper/zinc superoxide dismutase	III	30	174	CDS
gma-miR1509^a^	Glyma05g24110	elongation factor	IV	15	436	CDS
gma-miR1511^a^	Glyma10g05580*	60S ribosomal protein	II	28	1220	CDS
	Glyma13g19930*	60S ribosomal protein	III	28	1318	CDS
gma-miR1514^a^	Glyma11g35820#	NSF attachment protein	IV	13	651	CDS
	Glyma18g02590#	NSF attachment protein	IV	13	615	CDS
	Glyma07g05370	NAC family transcription factor	II	19	832	CDS
	Glyma16g01940	NAC family transcription factor	II	25	844	CDS
	Glyma16g01930	NAC family transcription factor	I	47	742	CDS
gma-miR1515^a^	Glyma12g00830	Autophagy protein	III	17	889	CDS
gma-miR1516^a^	Glyma04g42690	Disulfide isomerase	III	33	1016	CDS
gma-miR1522^a^	Glyma03g36390	FAD linked oxidase family protein	III	45	1826	5'UTR
gma-miR1523^a^	Glyma20g27950	polyubiquitinated protein	IV	114	864	CDS
gma-miR1530^a^	Glyma10g32330#	Auxin inducible transcription factor	III	24	79	3'UTR
	Glyma20g35280#	Auxin inducible transcription factor	III	24	445	CDS
	Glyma09g41100	expressed protein	II	20	1324	5'UTR
	Glyma02g28890	transketolase	III	104	67	CDS
gma-miR1536^a^	Glyma19g06340#	ribulose-1,5-bisphosphate carboxylase	III	108	795	5'UTR
	Glyma19g06370#	ribulose-1,5-bisphosphate carboxylase	III	108	668	5'UTR
	Glyma13g07610	ribulose-1,5-bisphosphate carboxylase	III	115	661	5'UTR

CDS: coding sequence; UTR: untranslated region; TP10M: transcripts per 10 million; Cleavage site: nucleotide number from 5' end of cDNA; Adjacent target genes with same ^# ^or * were matched by identical reads; ^a^legume or soybean specific miRNAs; ^b^MiRNA targets validated by RLM-5' RACE.

Among the 145 identified targets of known miRNAs, 114 targets (85%) belong to Class I and Class II, whereas 14 and 17 were classified into Classes III and IV, respectively (Table 3). Class I targets contained reads only from miRNA-directed cleavage, representing perfect data with no other contamination. A series of targets for known miRNAs, including gma-miR156, gma-miR159, gma-miR160, gma-miR164, gma-miR167, gma-miR169, gma-miR396, gma-miR398 and gma-miR1514, belong to this class (Tables 3, 4). More targets of soybean-specific miRNAs belong to Class III and Class IV when compared to those targets of conserved miRNAs.

**Table 4:** Identified targets of new miRNAs in soybean

miRNA	Target gene	Target annotation	Class	Abundance(TP10M)	cleavage site(nt)	Target site location
Soy_2	Glyma17g02170	F-box protein	II	15	67	CDS
Soy_3	Glyma07g39750#	PPR-containing protein	II	19	1633	CDS
	Glyma17g01050#	PPR-containing protein	III	19	1659	CDS
Soy_4	Glyma04g03110	oxidoreductase	IV	13	447	CDS
Soy_5	Glyma12g30680	60S ribosomal protein	III	17	643	5'UTR
Soy_7	Glyma16g25990	G-protein	II	15	1780	CDS
	Glyma19g37520	copper ion binding protein	IV	15	684	CDS
Soy_8	Glyma19g28990#	tubulin	III	17	920	CDS
	Glyma16g04420#	polyubiquitin protein	III	17	931	CDS
Soy_9	Glyma11g37920	HD-ZIP transcription factor	IV	19	629	CDS
Soy_10	Glyma19g22900	methyltransferase	IV	17	936	5'UTR
Soy_11	Glyma05g26750#	endomembrane protein	II	27	1407	CDS
	Glyma08g09740#	endomembrane protein	II	27	1416	CDS
	Glyma17g14370	ribosomal protein	IV	19	257	CDS
Soy_16	Glyma09g30740#	PPR-containing protein	I	14	616	CDS
	Glyma09g30680#	PPR-containing protein	IV	14	460	CDS
Soy_17	Glyma02g14400	expressed protein	III	15	955	5'UTR
Soy_19	Glyma19g35560#	Heat shock cognate protein	IV	47	282	CDS
	Glyma03g32850#	Heat shock cognate protein	IV	47	480	CDS
Soy_21	Glyma15g04010*	Transcription factor IIA	IV	14	694	CDS
	Glyma13g41390*	Transcription factor IIA	IV	14	1348	5'UTR
	Glyma19g03770	transferase protein	IV	14	746	CDS
	Glyma03g41900	bHLH family transcription factor	II	55	1184	CDS
Soy_22	Glyma19g41650	peptide chain release factor	IV	15	1258	5'UTR
Soy_25	Glyma05g33260	suppressor of gene silencing	II	30	555	CDS

CDS: coding sequence; UTR: untranslated region; TP10M: transcripts per 10 million; Cleavage site: nucleotide number from 5' end of cDNA; Adjacent target genes with same # or * were matched by identical reads.

### Validation of multiple genes matched by identical reads as targets of corresponding miRNAs

**Figure 5:**
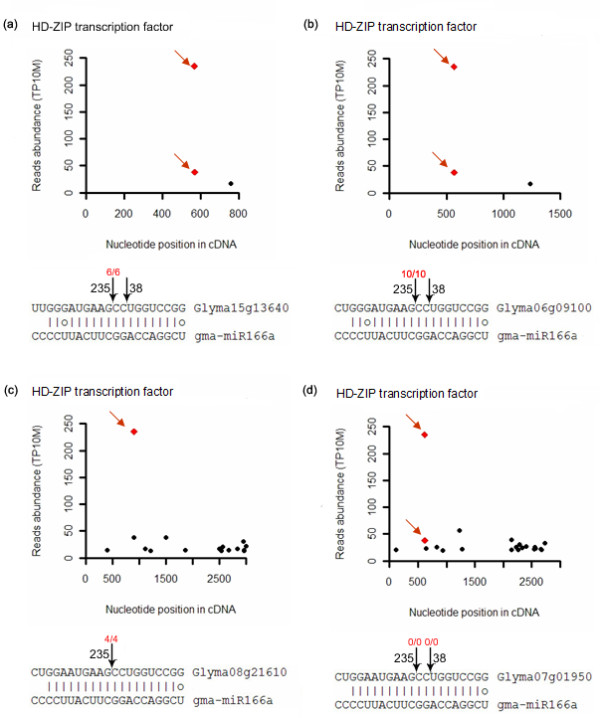
**Validation of gma-miR166 targets matched by identical reads**. The numbers of signatures along the sequences of targets were plotted. Red arrows indicate signatures produced by miRNA-directed cleavage. Black arrows above mRNA of targets indicate detected cleavage sites. Red numbers above the black arrows indicate cleavage probabilities (cleaved target vs total sequenced clones) through 5' RACE confirmation. Black numbers on the right or left side of each black arrow indicate detection abundance of reads. (a) Target cleavage signature, cleavage site in HD-ZIP transcription factor gene Glyma15g13640, and confirmation by RLM-5'RACE. (b) Target cleavage features in HD-ZIP transcription factor gene Glyma6g09100 and confirmation by RLM-5'RACE. (c) Cleavage features in HD-ZIP transcription factor gene Glyma08g21610 and confirmation by RLM-5'RACE. For (a), (b) and (c), only one of the two identified cleavage sites was further confirmed by RLM-5'RACE. (d) Gma-miR166 target HD-ZIP transcription factor gene (Glyma07g01950) from degradome sequencing could not be further confirmed by 5' RACE.

Because many soybean genes have multiple copies, some targets were matched by the same reads, as shown in Table 3. RLM-5' RACE experiments were applied to examine whether the targets mapped by the same reads were sliced by the same miRNA. For gma-miR166, 7 targets were matched by identical reads (Table 3). Among these, 4 HD-ZIP transcription factor genes were checked by 5' RACE (Figure 5). Three genes, Glyma13640, Glyma6g09100 and Glyma08g21610, were found to be cleaved by gma-miRNA166 after sequencing 6, 10 and 4 clones, respectively (Figure 5). One gene (Glyma07g01950) could not be confirmed to be cleaved by gma-miR166. Therefore, most of the genes with the identical signature could be regulated by the corresponding miRNA. By degradome sequencing, two cleavage sites were detected in 3 genes: Glyma13640, Glyma6g09100 and Glyma07g01950. However, only one cleavage site could be further validated by 5' RACE in Glyma13640 and Glyma6g09100 (Figure 5). The second cleavage site in these genes was not confirmed by 5' RACE, probably because of low frequencies.

Most miRNAs, especially conserved ones, could target several genes. The gma-miR396 had 21 target genes, and most of these could be grouped into Class I and Class II (Table 3). Every target cDNA had three regions: 5' UTR, CDS and 3' UTR. In animals, miRNA primarily binds to the 3' UTR of a gene to suppress translation. However, in plants, miRNA mainly silences gene expression through mRNA cleavage. In soybean, the cleavage site of the miRNA was usually located in the CDS of target genes (Table 3). Because genes with full-length cDNA represent only 5% of all predicted genes in the soybean database [7], the genes sliced by miRNA in the UTR region may not be detected because of incomplete information on gene sequences. However, miRNAs mainly cleave CDS of rice genes with relatively integrated gene sequences [13].

### Putative functions of annotated miRNA targets

Previous studies have found that miRNAs function in plants mainly by cleaving mRNA of transcription factors [26]. In this study, 82% of miRNA targets were transcription factors, a large number of which were auxin response factors, growth regulating factors and NAC transcription factors (Table 3). These factors may be involved in plant growth and/or responses to environmental changes. Most of the transcription factor gene targets belonged to Class I and Class II, indicating that miRNA was the key regulator of these genes.

**Figure 6:**
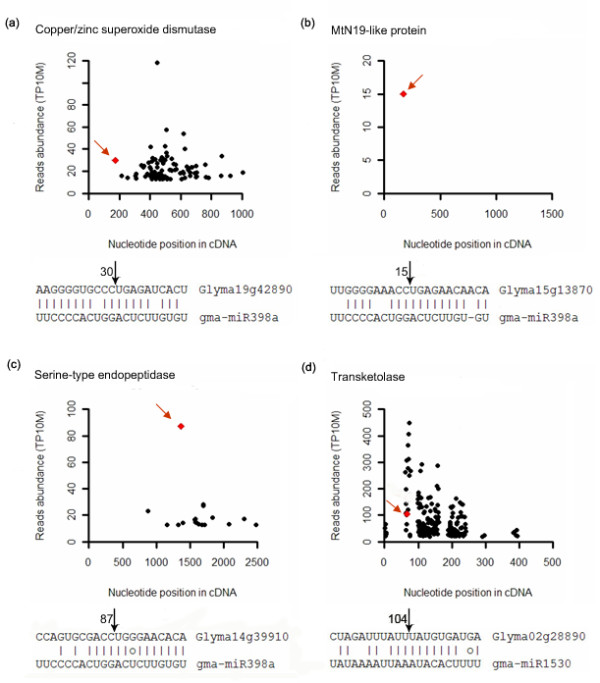
**Plot of signatures matched to miRNA targets and alignment of mRNA with miRNA**. (a) Cleavage features in target copper/zinc superoxide dismutase gene (Glyma19g42890) by gma-miR398a. (b) Cleavage features in target MtN19-like protein gene (Glyma15g13870) by gma-miR398a. (c) Cleavage features in target serine-type endopeptidase gene (Glyma14g39910) by gma-miR398a. (d) Cleavage features in target transketolase gene (Glyma02g28890) by non-conserved gma-miR1530. Other indications are as in Figure 5.

In most cases, targets of the same miRNA belong to the same gene family (Table 3); however, some miRNAs, such as gma-miR398, can target three types of genes, including copper/zinc superoxide dismutase, MtN19-like protein and serine-type endopeptidase (Figure 6a, b, c). In previous reports [13,27,28], sucrose-inducible miR398 was found to decrease expressions of two copper superoxide dismutase genes and a copper chaperone gene in Arabidopsis and rice. The copper superoxide dismutase gene was also found to be sliced by miR398 in soybean in our research (Figure 6a; Table 3). It seems likely that the role of miRNA398 in the regulation of copper superoxide dismutase genes is conserved among Arabidopsis, rice and soybean. Two other genes were also identified as gma-miR398 targets; one is a serine-type endopeptidase and the other is an MtN19-like protein induced by bruchin treatment [29] (Table 3; Figure 6b, c). Therefore, gma-miR398 may perform additional functions in soybean by targeting more genes.

Target genes of soybean- or legume-specific miRNAs primarily belong to Class III and Class IV, and these miRNAs regulate fewer targets than conserved miRNAs do (Table 3, the miRNAs denoted by ^a^). The target of gma-miR1530 was found to be a transketolase gene (Figure 6d), the product of which may participate in the Calvin cycle of photosynthesis. The Calvin cycle converts carbon dioxide into organic substances in plants; this process is known as carbon fixation. Therefore, the gma-miR1530 may regulate carbon assimilation in soybean. However, the gma-miR1530 was also identified from soybean root [8]. Two auxin induced transcription factors were also detected as targets of gma-miR1530, but their signature abundance was much lower (Table 3). Considering that the degradome library was constructed using developing soybean seeds, the gma-miR1530 may be responsible for the switch from carbon assimilation to energy metabolism during seed development by silencing the transketolase gene. However, it is possible that the gma-miR1530 targets may also participate in root development.

### Targets of new miRNAs from soybean

In addition to identification of the targets for known miRNAs (Table 3), targets of new miRNAs were investigated in this study (Table 4). The verification of miRNA targets provides further evidence for the existence of new miRNAs in soybean. We identified target genes for 15 new miRNAs (Table 4); these targets belonged mainly to Class III and Class IV, like the targets of soybean or legume-specific miRNAs (Table 3).

Unlike conserved miRNAs, the targets of new soybean miRNAs were not enriched in transcription factors (Table 4). Many target genes, such as G-protein and endomembrane protein, are likely involved in signal transduction, implying that the corresponding new miRNAs may participate in some specific developmental processes in soybean. Pentatricopeptide repeat proteins (PPR) were detected as the targets of Soy_3 and Soy_16. PPR-containing proteins perform functions at the post-transcriptional level in mitochondria and chloroplasts and are widely distributed in higher plants but absent in prokaryotes and archaebacteria [30,31]. They regulate gene expression in plant organelles through many processes, including RNA editing, cleavage and splicing. Soy_3 and Soy_16 may regulate plant organelle development by silencing genes encoding pentatricopeptide repeat-containing proteins.

**Figure 7:**
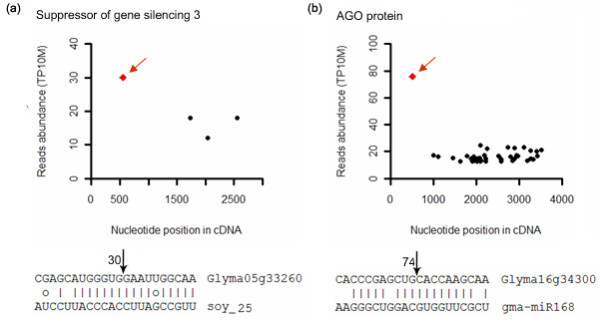
**SGS3 and AGO1 were sliced by soy_25 and gma-miR168, respectively**. (a) The gene for SGS3 (Suppressor of Gene Silencing 3) protein (Glyma05g33260) was identified as Soy_25 target by degradome sequencing. (b) The AGO gene (Glyma16g34300) was identified as the target of gma-miR168 by degradome sequencing.

Interestingly, the soybean homolog (Glyma05g33260) of Arabidopsis SUPPRESSOR OF GENE SLIENCING 3 (*AtSGS3*) was detected as the target of Soy_25 (Figure 7a). SGS3 was required for defense against virus infection through posttranscriptional gene silencing (PTGS) in plants [32-34]. *AtSGS3 *participates not only in mRNA degradation, but also in DNA methylation. Loss of function of *AtSGS3 *could reduce production of *trans*-acting siRNA in Arabidopsis [33]. The V2 protein of tomato yellow leaf curl virus could interact with tomato *SGS3 *to suppress RNA silencing for virus infection [33]. In addition to *SGS3*, soybean ARGONAUTE (*AGO*) proteins (Glyma16g34300 and Glyma09g29720), another important component in miRNA- or siRNA-mediated PTGS, were cleaved by conserved gma-miR168 (Figure 7b). Regulation of the *AGO *gene by miR168 was validated in Arabidopsis and rice [35].

### GO analysis of targets

**Figure 8:**
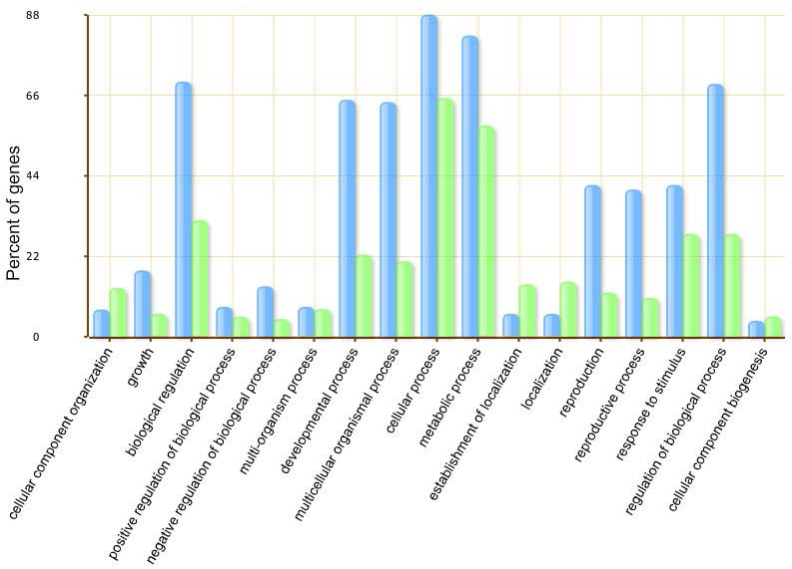
**GO analysis of targets of known and new miRNAs in this study**. Blue bars indicate the enrichment of miRNA targets in GO terms. Green bars indicate the percentage of total annotated soybean genes mapping to GO terms.

All targets regulated by soybean annotated miRNAs and new miRNAs identified in this study were subjected to AgriGO toolkit analysis to investigate gene ontology [36]. To date, 60,319 soybean genes have been annotated in the AgriGO database, and 159 soybean miRNA targets were recognized for GO analysis (Figure 8). As seen in Figure 8, more than 80% of these genes are involved in metabolic process, and reproduction-related genes were more enriched in miRNA targets than in soybean total genes. The enrichment of the genes involved in metabolic and reproductive processes may be consistent with the fact that both the small RNA and the degradome libraries were constructed from developing soybean seeds. The accumulation of dry matter for seed germination is the main task of developing seeds, and a large number of target genes may participate in these processes. The known and new miRNAs identified in this study may regulate expression of these target genes to control seed development and energy storage in soybeans.

## Discussion

As regulators of gene expression, miRNAs are widely present in animals and plants [37-50]. There are 243 and 511 miRNAs annotated in Arabidopsis and rice, respectively, according to the miRBase database [21,22]. Soybean is an ancient polyploid (paleopolyploid) crop plant, with a more complex and larger genome than Arabidopsis and rice [7]. In total, 207 soybean miRNAs were annotated in the miRBase database. In this study, 26 new miRNAs were identified in soybean by deep sequencing and validated by experimental approaches. Functional elucidation and target analysis of the conserved and non-conserved miRNAs could yield more clues to the different regulations of gene expressions between species.

We further studied the target genes of the miRNAs by degradome library sequencing. Unlike other species studied by degradome sequencing [11-16], in the soybean genome, only 40% of Solexa reads could be mapped to a single position. Previous reports showed soybean to be an ancient polyploidy, and the genome duplicated twice, 59 and 13 million years ago [7]. Most genes have multiple copies in the genome. In addition, the tags from degradome sequencing have only 20 or 21 nt. Therefore, the proportion of single mapped reads was not as high as that in other degradome sequencing. Extending the length of sequencing tag may improve this proportion.

Because of the high throughput of deep sequencing, a large number of reads that were not miRNA-directed cleavage products were detected in Class III and Class IV. These cleavages may have been derived from cleavage by other unidentified soybean miRNAs. Conserved miRNAs silence more targets than soybean-specific miRNAs. It is possible that conserved miRNAs play a key role in universal mechanisms of regulation in different plant species;, however, soybean-specific miRNAs may function only in regulation of gene expression during legume- or soybean-specific processes. Although the conserved miRNAs mainly regulate genes encoding transcription factors, soybean-specific miRNAs regulate various types of genes, suggesting a new feature of miRNA regulation in soybeans.

As the small RNA library was prepared from soybean developing seeds, the miRNAs with detected target genes should take part in regulation of seed development. Although most of the soybean genes were not annotated clearly, some targets related to seed development were identified in this study. The soybean seed is a storage organ, containing significant amounts of lipid and protein. Energy metabolism is very active during development of seeds, especially in chloroplasts. In the early stages of soybean seed development, photosynthesis occurs in seed chloroplasts. Subsequently, lipid accumulation becomes the major function of chloroplasts in seeds. Genes encoding transketolase and carboxylase in these processes were identified as gma-miR1530 and gma-miR1536 targets, respectively. Genes encoding PPR-containing proteins, which regulate gene expression in mitochondria and chloroplasts, were also regulated by some miRNAs. These miRNAs may affect conversion between photosynthesis and lipid accumulation in seeds by regulating genes related to metabolism and chloroplast development. Moreover, comparison of miRNA abundance in seeds and other organs of soybeans should uncover those miRNAs specifically expressed in seeds. Identification of the corresponding target genes and study of their roles will elucidate possible functions of miRNAs and target genes in relevant processes of seed development.

Only a few annotated conserved miRNAs were found to have no soybean target genes; however, many non-conserved miRNAs did not appear to silence any targets. MiRNAs regulate gene expression not only by mRNA cleavage but also by translation repression. The miRNAs with no detected targets may silence genes by repressing translation. However, we could not obtain information about translation repression by miRNA through degradome sequencing. Other methods may be used to detect such a possibility, e.g., co-expression of miRNA and the predicted target in *N. benthamiana *leaves [13]. Some non-conserved miRNAs are hard to detect because of low abundance or spatial expression pattern. To get more integrated information on miRNA targets, degradome libraries from different tissues, organs and different developmental stages should be constructed. Additionally, some miRNAs also function in methylation of genomic DNA or histones. More attention should be paid to the mechanism of methylation via miRNA to clarify other functions of miRNA.

In higher plants, miRNAs function mainly through silencing related gene expression. Identification of miRNA targets will help us to understand the biological effects of miRNA. By deep sequencing of a degradome library, we identified a large number of target genes regulated by corresponding miRNAs (Table 3, 4). These targets contained not only conserved families of miRNA target genes, such as *MYB, ARF, NAC, GRF *and *TCP*-type transcription factor gene families, but also non-conserved target genes, such as G-protein, SGS3 and F-box protein. The conserved targets may participate in various aspects of plant development and stress responses as in other plants and may help us to understand evolutionary relationships between soybean and other plants. Global identification of non-conserved targets provides useful information to explore the new functions of miRNAs in soybean. The regulation of *SGS3 *by miRNA was not found in previous studies. Further study of the relationship between *SGS3 *and the new miRNA Soy_25 should reveal the function of this pair in regulation of miRNA biogenesis and/or seed development in soybean and other plants.

## Conclusions

In our study, a small RNA library and a degradome library were constructed from developing soybean seeds for deep sequencing. We identified 26 new miRNAs in soybean by bioinformatic analysis and experimental tests. The miRNA star sequences of 38 known miRNAs and 8 new miRNAs were also discovered, providing additional evidence for the existence of miRNAs. Degradome sequencing as a high-throughput approach for miRNA target detection was applied to identify miRNA targets in soybean. In total, 145 and 25 genes were identified as targets of annotated miRNAs and new miRNAs, respectively. Construction of degradome libraries from different developmental stages of seeds should reveal more targets of soybean miRNAs. Overall, global identification of soybean miRNA targets in this study provides more information about the regulatory network of miRNAs in soybean, and it will advance our understanding of miRNA functions during seed development.

## Methods

### Plant material and RNA isolation

Soybean (*Glycine max*) seeds of cultivar Heinong44 were directly planted in the Experimental Station of the Institute of Genetics and Developmental Biology, Chinese Academy of Sciences, in Beijing in May. Seeds from soybeans 15 days after flowering (DAF) were collected and quickly frozen in liquid nitrogen. Total RNA was isolated from seeds using TRIzol reagent (Invitrogen) according to the manufacturer's instructions.

### Small RNA library and degradome library construction

After total RNA isolation, low molecular weight RNAs were isolated as described previously, with some modification [51]. By polyacrylamide gel electrophoresis, the small RNAs (~17-27 nt) were purified from 100 μg of total RNA and ligated to a 5' RNA adapter and a 3' RNA adapter. A reverse transcription reaction followed by low cycle PCR was performed to obtain sufficient product for SBS sequencing. PCR products were collected by gel purification and sequenced by Solexa technology.

The soybean seed degradome library was constructed as previously described [18,19]. In brief, poly(A) RNA was extracted from 200 μg of total RNA using the Oligotex kit (Qiagen). A 5' RNA adapter containing a MmeI recognition site was ligated to the poly(A) RNA possessing a 5'-phosphate, by T4 RNA ligase (Ambion), and the ligated products were repurified using the Oligotex kit. Five PCR cycles were then performed to amplify the reverse transcription products. The PCR products were digested with MmeI and ligated to a 3' double DNA adapter. The ligation products were amplified by 20 PCR cycles and gel-purified for SBS sequencing.

The small RNA library and degradome library sequencing data were available under NCBI-GEO accession no. GSE25260.

### Bioinformatic analysis of sequencing data

Small RNA reads and degradome reads were both generated from an Illumina Genome Analyzer II. The row data were preprocessed by the Fastx-toolkit pipeline to remove low quality reads and clip adapter sequences. As for the small RNA library, small RNAs ranging from 18-25 nt were collected and mapped to the soybean genome using SOAP2 [19]. The unique RNA sequences that perfectly matched the genome were subjected to subsequent analysis. RNA reads showing sequences identical to known miRNAs from the miRBase database [21,22] were picked up as the miRNA dataset of soybean. Sequences matching noncoding rRNA, tRNA, snRNA and snoRNA in the Rfam database were removed. Reads overlapping with exons of protein-coding genes were excluded to avoid mRNA contamination. The remaining sequences were considered for prediction to find new miRNAs.

As for the degradome library, only 20-21 nt sequences with high quality were collected for subsequent analysis. The raw sequences were first normalized to "reads per 10 million" (RP10M). The distinct reads that perfectly matched soybean cDNA sequences remained. The 15 nt of sequence upstream and downstream of the 5' end of matched reads were extracted to constitute 30-nt sequence tags for searching corresponding miRNA. The CleaveLand pipeline [18] was used to align the 30 nt sequence to soybean known miRNAs from miRBase and our newly identified miRNAs. All alignments with scores up to 7 and no mismatches at the cleavage site (between the 10^th ^and 11^th ^nucleotides) were considered candidate targets.

### Prediction of new miRNAs

As miRNA precursors have a characteristic hairpin structure, 150 nt of the sequence flanking the genomic sequences of small RNAs was extracted. The MIREAP pipeline was then used to analyze their structural features to identify new miRNA candidates (https://sourceforge.net/projects/mireap/). The resulting structures, with minimal matched nucleotide pairs of miRNA and miRNA* exceeding 16 nt and with maximal size differences of miRNA and miRNA* up to 4 nt, were retained as new miRNA candidates. The filtered pre-miRNA sequences were folded again using MFOLD and checked manually [23].

### Stem-loop RT-PCR

Reverse transcription reactions were performed using total RNA from soybean seeds as previously described [25]. All primers involved in stem-loop RT-PCR are listed in Table S2 in Additional File 2. The reactions contained 25 ng of RNA samples, 50 nM stem-loop RT primer (Invitrogen), 1 × RT buffer, 0.25 mM of each dNTP (Takara), 5 U/μl SuperScript II reverse transcriptase (Invitrogen) and 0.25 U/μl RNase Inhibitor (Invitrogen). The 10 μl reactions were incubated in a Biometra TProfessional Thermocycler in a 96-well plate for 30 min at 16°C, 30 min at 42°C and 5 min at 85°C and then held at 4°C.

PCR was performed using diluted cDNA products. The reactions were incubated in a Biometra TProfessional Thermocycler for 5 min at 95°C, followed by 40 cycles of 15 sec at 94°C, 30 sec at 60°C and 30 sec at 72°C. All reactions were run in duplicate. The PCR products were detected by gel electrophoresis.

### RLM-5' RACE

Total RNA (200 μg) from soybean seeds was used to purify mRNA using the Oligotex kit (Qiagen). 5' RNA adaptor (5'-CGACUGGAGCACGAGGACACUGACAUGGACUGAAGGAGUAGAAA-3') was ligated to the purified mRNA by T4 RNA ligase (Ambion), followed by a reverse transcription reaction. The reverse transcription product was amplified using 5' RNA adaptor primer (5'-GCACGAGGACACTGACATGGACTGA-3') and gene specific primers for 30 cycles of PCR. Twenty-five cycles of PCR were further performed with the above PCR product as templates, using a nested gene specific primer (5'-GGACACTGACATGGACTGAAGGAGTA-3') and an adapter primer. The final PCR product was detected by gel electrophoresis and extracted for sequencing.

## Abbreviations

miRNA: microRNA; pre-miRNA: miRNA precursor; miRNA*: miRNA star; poly(A) RNA: Polyadenylated RNA; RT: Reverse-transcription.

## Authors' contributions

QXS performed the bioinformatics analysis, analyzed the Solexa data, conducted the experiments and drafted the initial manuscript. YFL prepared the materials and RNAs. XYH was involved in Solexa sequencing. WKZ and BM contributed to the experimental design and analysis. SYC and JSZ conceived the study, obtained the funding, analyzed the data and finished the final manuscript. All authors read and approved the final manuscript.

## Supplementary Material

Additional file 1 **New miRNAs identified in soybean developing seeds**. Mature sequences, star sequences and precursor sequences of miRNAs. The numbers of miRNAs detected by small RNA library sequencing are also included. Click here for file

Additional file 2 **Stem-loop RT-PCR Primers**. All primers used in stem-loop RT-PCR. Click here for file
